# Functional Integrity of Executive Control Network Contributed to Retained Executive Abilities in Mild Cognitive Impairment

**DOI:** 10.3389/fnagi.2021.710172

**Published:** 2021-11-26

**Authors:** Wan Liu, Li Liu, Xinxin Cheng, Honglin Ge, Guanjie Hu, Chen Xue, Wenzhang Qi, Wenwen Xu, Shanshan Chen, Run Gao, Jiang Rao, Jiu Chen

**Affiliations:** ^1^Department of Rehabilitation, The Affiliated Brain Hospital of Nanjing Medical University, Nanjing, China; ^2^Institute of Brain Functional Imaging, Nanjing Medical University, Nanjing, China; ^3^Institute of Neuropsychiatry, The Affiliated Brain Hospital of Nanjing Medical University, Nanjing, China; ^4^Department of Radiology, The Affiliated Brain Hospital of Nanjing Medical University, Nanjing, China; ^5^Department of Neurology, The Affiliated Brain Hospital of Nanjing Medical University, Nanjing, China

**Keywords:** mild cognitive impairment, executive function, executive control network, functional connectivity, fractional amplitude of low-frequency fluctuation

## Abstract

**Background:** Mild cognitive impairment (MCI) is considered to be a transitional state between normal aging and Alzheimer's dementia (AD). Recent studies have indicated that executive function (EF) declines during MCI. However, only a limited number of studies have investigated the neural basis of EF deficits in MCI. Herein, we investigate the changes of regional brain spontaneous activity and functional connectivity (FC) of the executive control network (ECN) between high EF and low EF groups.

**Methods:** According to EF composite score (ADNI-EF) from the Alzheimer's Disease Neuroimaging Initiative (ADNI), we divided MCI into two groups, including the MCI-highEF group and MCI-lowEF group. Resting-state functional MRI was utilized to investigate the fractional amplitude of low-frequency fluctuation (fALFF) and ECN functional connectivity across 23 healthy controls (HC), 11 MCI-highEF, and 14 MCI-lowEF participants. Moreover, a partial correlation analysis was carried out to examine the relationship between altered fALFF or connectivity of the ECN and the ADNI-EF.

**Results:** Compared to HC, the MCI-highEF participants demonstrated increased fALFF in the left superior temporal gyrus (STG), as well as decreased fALFF in the right precentral gyrus, right postcentral gyrus, and left middle frontal gyrus (MFG). The MCI-lowEF participants demonstrated increased fALFF in the cerebellar vermis and decreased fALFF in the left MFG. Additionally, compared to HC, the MCI-highEF participants indicated no significant difference in connectivity of the ECN. Furthermore, the MCI-lowEF participants showed increased ECN FC in the left cuneus and left MFG, as well as decreased ECN functional connectivity in the right parahippocampal gyrus (PHG). Notably, the altered fALFF in the left MFG was positively correlated to ADNI-EF, while the altered fALFF in cerebellar vermis is negatively correlated with ADNI-EF across the two MCI groups and the HC group. Altered ECN functional connectivity in the right PHG is negatively correlated to ADNI-EF, while altered ECN functional connectivity in the left cuneus is negatively correlated to ADNI-EF across the three groups.

**Conclusions:** Our current study demonstrates the presence of different patterns of regional brain spontaneous activity and ECN FC in the MCI-highEF group and MCI-lowEF group. Furthermore, the ECN FC of the MCI-highEF group was not disrupted, which may contribute to retained EF in MCI.

## 1. Introduction

Mild cognitive impairment (MCI) is a transitional state between normal aging and Alzheimer's dementia (AD) (Bohlken et al., 2019; Thomas et al., 2019). Among patients with MCI, it has been well-established that patients with memory impairment (amnestic MCI, aMCI) are at a high risk of developing AD (Park et al., 2017; Thomas et al., 2017). Although memory deficits during disease progression have been widely studied and represent a benchmark of a probable AD diagnosis (Scheltens et al., 2018), more recent research has investigated executive function (EF) decline during MCI, which is also referred to as the preclinical stage of AD (Chang et al., 2009; Ewers et al., 2014; Kirova et al., 2015). EF has the ability required to plan, organize, operate on working memory, as well as switch between tasks (Bettcher et al., 2016). A recent study has developed a composite measure of EF, ADNI-EF, utilizing neuropsychological data from the Alzheimer's Disease Neuroimaging Initiative (ADNI), and has reported that ADNI-EF is a major predictor of transition from MCI to AD (Gibbons et al., 2012a). It has been concluded that poor EF in patients with MCI is characterized by very early cognitive decline in the initial course of AD, and indicates a transition from MCI to AD. Thus, it is greatly significant to clarify neuropathological mechanism of EF impairment and identify features that can predict their progression to AD.

While a large number of studies have examined the neuropathological factors related to MCI memory impairment (Perrotin et al., 2015; Terry et al., 2015; Vijayakumari et al., 2020), only a limited number of studies have examined the neural basis of EF deficits in MCI. Therefore, these limited studies have explored neural mechanisms of EF decline in patients with MCI from different aspects of brain morphology, metabolism, and network function. A study from the perspective of brain morphology has demonstrated that among patients with aMCI, the atrophic brain areas associated with decreasing of EF are located in the frontal and temporal cortex and that the atrophy of the right inferior frontal gyrus is more closely related to decreasing EF (Zheng et al., 2014). However, results from the metabolic point of view are not consistent with this, and the results indicated that EF impairment in aMCI is related to cerebral glucose metabolic abnormalities in the anterior cingulate cortex (ACC) and posterior cingulate cortex (PCC) (Yoon et al., 2020). Damage to the EF in MCI is not only related to the abnormality of local brain structure and brain metabolism but also to the brain network. A functional MRI (fMRI) study validated that the presence of increased connectivity of the ACC and dorsal lateral prefrontal cortex (DLPFC) in the ECN is positively correlated to EF in aMCI (Wu et al., 2014). Similarly, a diffusion tensor imaging (DTI) study demonstrated that MCI with high EF has a larger network size, density, and clustering coefficient (Farrar et al., 2018). However, results from fewer previous studies were not entirely consistent, and little was known about the changes in both spontaneous brain activity and brain functional networks.

In recent years, resting-state fMRI (rs-fMRI), attracted significant research interest in studying neural mechanisms of cognitive dysfunction (d'Ambrosio et al., 2020; Lee et al., 2020; Li et al., 2020). Among them, the fractional amplitude of low-frequency fluctuation (fALFF) was utilized to reliably measure the intensity of brain activity (Shu et al., 2020; Li et al., 2021). As it is a whole-brain data-driven method with high test-retest reliability, the fALFF has been chosen to carry out many studies among patients with MCI (Qiu et al., 2019; Yu et al., 2019; Zeng et al., 2019). In addition, it is well-known that EF requires several distinct brain regions that work together to perform complex tasks effectively (Farrar et al., 2018). Therefore, EF is suitable for network analysis. Moreover, the ECN comprising the main brain regions in the medial frontal cortex, ACC, DLPFC, is involved in top-down, attention-dependent EF such as cognitive control and response inhibition (Chen et al., 2008; Brown et al., 2019).

Therefore, according to ADNI-EF, patients with MCI were divided into two groups, including the high EF group and the low EF group. The objective of this current study is to investigate changes in regional brain spontaneous activity and FC of ECN between the two groups, as well as to further investigate the relationship between changes in the brain activity or FC of the ECN and EF. We hypothesized that there are different altered brain spontaneous activity and FC of ECN between the two groups, and changes of the low EF group may be more significant and similar to the pathological patterns of AD.

## 2. Materials and Methods

In total, 109 subjects participated in the current study, which included 84 patients with MCI and 25 healthy controls (HC). All participants were chosen from the in-house database, the Nanjing Brain Hospital-Alzheimer's Disease Spectrum Neuroimaging Project (NBH-ADsnp) (Nanjing, China), which is continuously updated. The details of the NBH-ADsnp-related information are provided in Supplementary Material. The diagnostic and exclusion criteria of MCI and HC were in accordance with our previous studies (Xue et al., 2019; Wang et al., 2021b). This study was granted approval by the responsible Human Participants Ethics Committee of the Affiliated Brain Hospital of Nanjing Medical University (Nos. 2018-KY010-01 and 2020-KY010-02), located in Nanjing, China. All participants were granted written informed consent prior to participation.

### 2.1. Neuropsychological Assessments

All participants underwent a comprehensive and standard assessment of neurocognitive function, including general cognitive function, information processing speed, episodic memory, visuo-spatial function, and EF. Details regarding each of these assessments were consistent with previous studies (Xue et al., 2019; Wang et al., 2021a).

### 2.2. Grouping

First, the EF composite score (ADNI-EF) of 84 patients with MCI was calculated according to the model provided by the ADNI website (http://adni.loni.usc.edu/). This model contains a WAIS-R Digit Symbol Substitution, Digit Span Backwards, Trails A and B, Category Verbal Fluency Test (CVFT), and Clock Drawing (Gibbons et al., 2012b). In our study, the average score was −0.91, with a SD of 0.46. Individuals with MCI with high executive abilities (MCI-highEF participants) were classified as being one SD above the group mean EF score, which led to 13 participants having a score above −0.45. Individuals with cognitive impairment that have low executive abilities (MCI-lowEF participants) were categorized as being one standard deviation below the group mean, leading to 15 participants with a score below −1.37. Similarly, 25 HCs were matched with 28 MCI participants (13 MCI-highEF participants and 15 MCI-lowEF participants). However, two MCI-highEF participants, one MCI-lowEF participant, and two HCs were excluded due to excessive head movement (> 3*mm or* > 3°). Finally, 25 patients with MCI were enrolled, which included 11 MCI-highEF participants, 14 MCI-lowEF participants, and 23 HCs.

### 2.3. MRI Data Acquisition

The detailed parameters of MRI acquisition of NBH-ADsnp were summarized in Supplementary Material.

### 2.4. Image Preprocessing

Data processing was conducted utilizing Data Processing Assistant for Resting-State fMRI (DPARSF 4.4, http://www.restfmri.net) based on the Matlab2013b platform. The first 10 volumes of functional images were removed for each subject. Then, the remaining images were corrected using slice-timing and realignment, accounting for head motion, normalized to standard space using DARTEL, resampled to a 3 × 3 × 3*mm*^3^ voxel size, regress nuisance variable, and spatially smoothed with 4 mm full width at half maximum (FWHM). The nuisance variables include 24 motion parameters (six head motion parameters, six head motion parameters one time point before, and the 12 corresponding squared items), a global signal, a white matter signal, and a cerebrospinal fluid signal. Finally, we carried out filtering band-pass (0.01-0.08 Hz) (Chen et al., 2016) prior to calculating seed-based functional connectivity (FC), and after calculating fALFF. In addition, participants with excessive head motion (cumulative translation or rotation >3.0 mm or 3.0) were excluded(Chen et al., 2020; Wang et al., 2021a).

### 2.5. fALFF Analysis

After data preprocessing, we carried out fALFF for each scan. The fast Fourier transform helped transform the time series of each voxel to the frequency domain in order to obtain the power spectrum. Then, the square root of the power spectrum was calculated. The fALFF was attained using the ratio of the power spectrum in a given frequency band (0.01–0.08 Hz) to total power in the entire detectable frequency range (Zou et al., 2008). Finally, the fALFF value of each voxel was divided using the global mean value in order to decrease global effects across participants.

### 2.6. FC Analysis

A seed-based FC analysis was carried out to examine the alteration of ECN. Seed region of interest (ROI) by drawing the 6-mm spheres located in the right DLPFC (MNI space: 48, 12, 34) was determined by converging data from previous studies (Smith et al., 2009; Wang et al., 2016). The DLPFC was consistently considered to be a key region within the ECN. Individual mean time series were extracted based on the coregistered seed region as the reference time series. The correlation analyses were conducted on the seed region and whole brain in a voxel-wise manner. The correlation coefficients of each voxel were normalized to Z-scores using Fisher's r-to-z transformation. Therefore, an entire brain Z-score map was developed for each subject for subsequent statistical analyses.

### 2.7. Statistical Analyses

The ANOVA was conducted to compare the demographics, neuropsychological assessment, and head rotation parameters among the three groups, except for gender (chi-square test). The two-sample *t*-test was used for *post-hoc* comparisons. The *p*-value was set as < 0.05 for significant differences. A one-way analysis of covariance (ANCOVA) was utilized for comparison of the differences of FC in ECN and fALFF among HC, MCI-highEF, and MCI-lowEF participants. We used demographic data (age, gender, and education level), and gray matter volume as covariables. As suggested in the previous study, a non-parametric permutation test was able to precisely control the false positive rate in cluster-level inference (Qi et al., 2010). Therefore, we set the permutation times at 1,000. The corrected *p* < 0.01 (fALFF results) or *p* < 0.05 (FC results) was used for statistical significance and cluster size >50 voxels (1,350 *mm*^3^) was applied for multiple comparisons at the voxel level. Then, the two-sample *t*-test was used for *post-hoc* comparisons, and the mask resulted from ANCOVA analyses after controlling the effects of demographic data (age, gender, and education level), and gray matter volume. We also set significance with the threshold free cluster enhancement and family-wise error (TFCE-FWE) corrected cluster p < 0.05 and the cluster size > 10 voxels (270 *mm*^3^). Finally, FCs or fALFF of significantly altered regions were extracted and later utilized for correlation analyses. The partial correlation analyses were carried out to reveal relationships between the altered fALFF or FCs and ADNI-EF after adjusting for the effects of age, gender, and education level. Because of the relatively small sample size, we did not correct the correlation analysis results for multiple comparisons in order to better present the results. The statistical significance was determined by an uncorrected p < 0.05.

## 3. Results

### 3.1. Demographic and Neuropsychological Characteristics

In parallel, EF of the MCI-lowEF group was lower compared to the MCI-high group (*p* < 0.05) (Table 1). We found no significant differences in gender, head motion parameters (Supplementary Table 1) or education level were observed between the MCI-highEF group, MCI-lowEF group, and the HC group (all *p* >0.05). The MCI-lowEF group was older, compared to the HC subjects (70 ± 7.47 vs. 60.96 ± 9.45, *p* < 0.05) and MCI-highEF group (70 ± 7.47 vs. 60 ± 7.47, *p* < 0.05). In comparison to HCs, MCI-highEF patients only showed significantly decreased MoCA and ADNI-EF scores, while MCI-lowEF patients exhibited significantly reduced MMSE, MoCA, and ADNI-EF (all *p* < 0.05). In addition, compared to the MCI-highEF group, the MCI-lowEF group demonstrated a significant decline in MMSE, MoCA, and ADNI-EF (all *p* < 0.05) (Table 1).

**Table 1:** Demographics and clinical measures of patients with mild cognitive impairment (MCI) and healthy controls (HC).

	**HCs (*n* = 23)**	**MCI-highEF (*n* = 11)**	**MCI-lowEF (*n* = 14)**	**F(**χ^2^**)**	***P***
Age (years)	60.96 ± 9.45	60.00 ± 6.68	70.00 ± 7.47^*b, c*^	6.27	0.004^*^
Gender (M/F), n	8/15	2/9	4/10	1.00	0.608
Education (years)	12.70 ± 2.24	12.18 ± 2.32	10.68 ± 3.16	2.75	0.075
MMSE	28.430 ± 1.56	27.73 ± 1.01	26.36 ± 1.45^*b, c*^	9.30	<0.001^*^
MoCA	26.65 ± 1.70	23.91 ± 1.70	22.21 ± 2.46^*a, b, c*^	9.30	<0.001^*^
ADNI-EF	-0.11 ± 0.36	−0.33 ± 0.18	−1.60 ± 0.20^*a, b, c*^	121.81	<0.001^*^

*Data is represented by mean ± SD unless otherwise indicated. M, male; F, female; MMSE, Mini-Mental State Examination; MoCA, Montreal Cognitive Assessment*.

* *Significant differences were found among HC, MCI-highEF, and MCI-lowEF subjects. Most p-values were obtained using ANOVA, except for gender (chi-square test). Comparisons of each paired group were conducted to further reveal the source of ANOVA difference (a: MCI-highEF vs. HCs; b: MCI-lowEF vs. HCs; c: MCI-lowEF vs. MCI-highEF)*.

### 3.2. Comparison of fALFF Between the Patients With MCI and the HC

**Figure 1:**
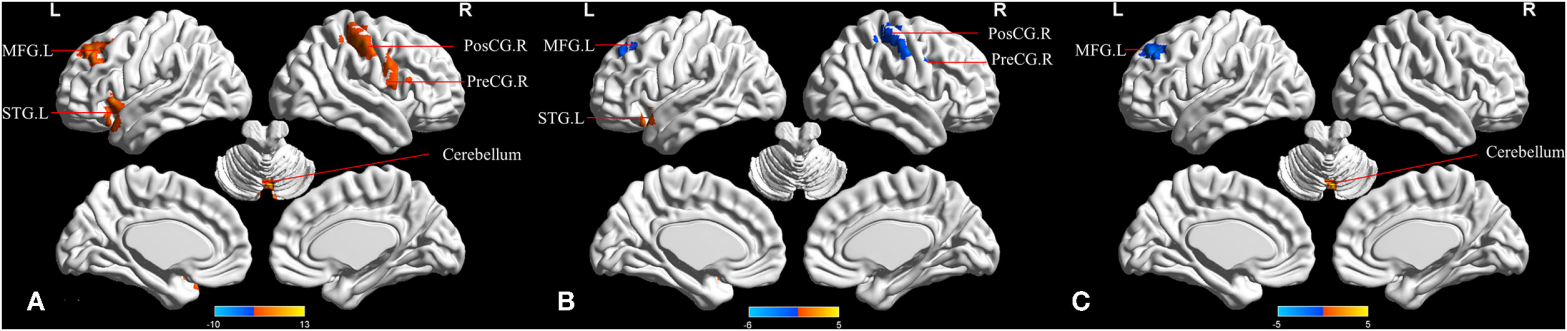
Regions that demonstrate between-group differences in the fractional amplitude of low-frequency fluctuation (fALFF). **(A)** Regions with significant differences across the three groups in fALFF (one-way analysis of covariance (ANCOVA), cluster *p* < 0.01, cluster size >50 voxels). **(B)** Regions with significant differences of MCI-highEF group vs. HC in fALFF [two-sample *t*-test; cluster *p* < 0.05; cluster size >10 voxels; TFCE-FWE (family-wise error) corrected]. **(C)** Regions with significant differences of the MCI-lowEF group vs. HC in fALFF (two-sample *t*-test; cluster *p* < 0.05; cluster size >10 voxels; TFCE-FWE corrected). MFG.L, left middle frontal gyrus; PosCG.R, right postcentral gyrus; PreCG.R, right precentral gyrus; STG.L, left superior temporal gyrus.

When comparing the three groups, the ANCOVA analysis demonstrated significantly altered fALFF across the five brain regions among the groups, including in the cerebellar vermis, left superior temporal gyrus (STG), right precentral gyrus, left middle frontal gyrus (MFG), and right postcentral gyrus (Figure 1A and Table 2). Compared to HC, the MCI-highEF participants had significantly higher fALFF in the left STG, and decreased fALFF in the right precentral gyrus, right postcentral gyrus, and left MFG (Figure 1B and Table 2). The MCI-lowEF participants also showed significantly increased fALFF in the cerebellar vermis and decreased fALFF in the left MFG (Figure 1C and Table 2). Compared to the MCI-highEF participants, the MCI-lowEF participants demonstrated no significant differences in fALFF in these brain regions.

**Table 2:** The differences in the fractional amplitude of low-frequency fluctuation (fALFF) among the three groups.

**Region (aal)**	**Peak MNI coordinate**			**F/t**	**Cluster number**
	**x**	**y**	**z**		
ANCOVA					
Cerebellar vermis	6	−72	−24	12.697	57
Left superior temporal gyrus	−39	21	−18	9.852	89
Right precentral gyrus	57	9	39	11.033	77
Left middle frontal gyrus	−24	36	42	11.557	100
Right postcentral gyrus	54	−30	48	10.570	124
MCI-highEF>HC					
Left superior temporal gyrus	−39	18	−18	4.782	35
MCI-highEF < HC					
Right precentral gyrus	54	6	39	−5.359	16
Right postcentral gyrus1	60	−12	42	−4.354	12
Right postcentral gyrus2	36	−33	60	−5.025	59
Left middle frontal gyrus	−30	30	48	−4.323	27
MCI-lowEF>HC					
Cerebellar vermis	3	−72	−15	4.656	24
MCI-lowEF < HC					
Left middle frontal gyrus	−24	36	42	−4.782	48

### 3.3. Comparison of FC Between the Patients With MCI and the HC

**Figure 2:**
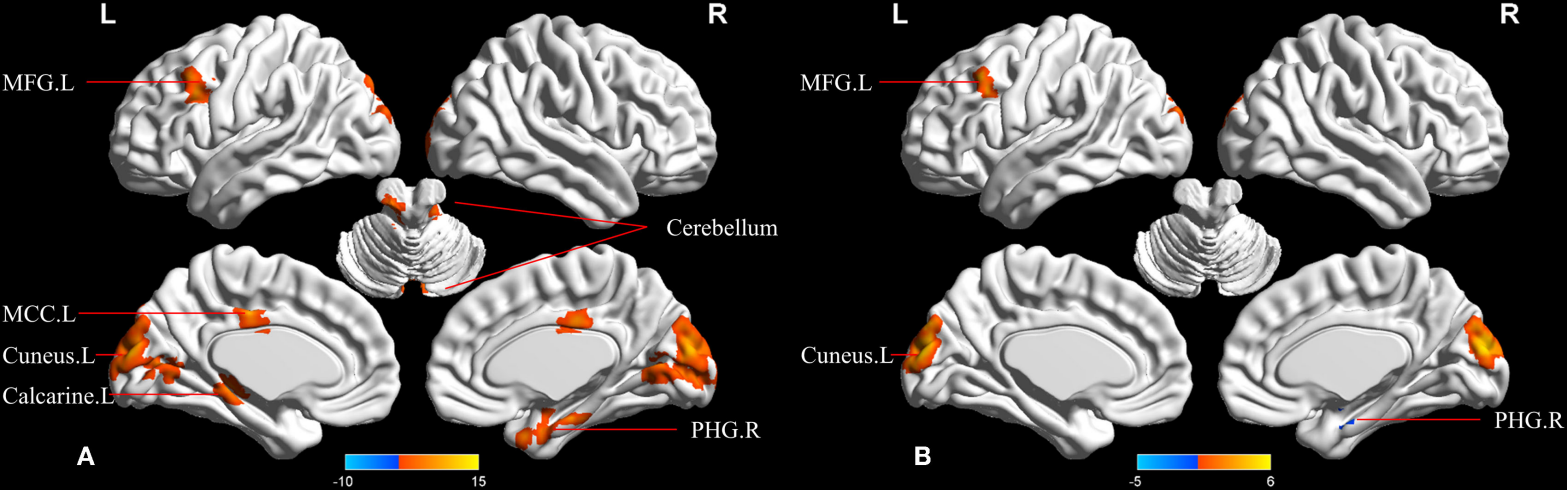
Regions showing between-group differences in functional connectivity (FC) of the executive control network (ECN). **(A)** Regions with significant differences among the three groups in FC of ECN. (one-way ANCOVA; cluster *p* < 0.05; cluster size >50 voxels). **(B)** Regions with significant differences of MCI-lowEF group vs. HC in FC of ECN (two-sample *t*-test; cluster *p* < 0.05; cluster size >10 voxels; TFCE-FWE corrected). MCC.L, left middle cingulum cortex; MFG.L, left middle frontal gyrus; PHG.R, right parahippocampal gyrus.

In the ECN, upon the comparison of the three groups, the ANCOVA analysis demonstrated the seven significantly altered FCs between the right DLPFC and brain regions among the groups, including the left cerebelum_crus, right parahippocampal gyrus (PHG), left cerebelum_4_5, left calcarine, left MFG, and left middle cingulum (Figure 2A and Table 3). Compared to the HC, the MCI-highEF participants demonstrated no significant difference in the connectivity of the ECN. In addition, the MCI-lowEF participants indicated significantly increased FC in the left cuneus, left MFG, and decreased FC in the right PHG (Figure 2B and Table 3). Compared to the MCI-highEF participants, the MCI-lowEF participants demonstrated no significant difference in FC of the ECN.

**Table 3:** The differences in functional connectivity (FC) of executive control network (ECN) among the three groups.

**Region (aal)**	**Peak MNI coordinate**			**F/t**	**Cluster number**
	**x**	**y**	**z**		
**ANCOVA**					
Left cerebelum_crus	−3	−78	−33	7.962	52
Right parahippocampal	24	−6	−24	9.787	74
Left cerebelum_4_5	−9	−36	−9	11.632	93
Left cuneus	0	−90	18	14.376	409
Left calcarine	−12	−78	9	6.252	54
Left middle frontal gyrus	−42	18	33	10.072	71
Left middle cingulum	−3	−21	39	9.708	52
**MCI-lowEF>HC**					
Left cuneus	0	−93	15	5.515	189
Left middle frontal gyrus	−42	21	36	4.634	22
**MCI-lowEF < HC**					
Right parahippocampal	24	−9	−24	−4.698	10

### 3.4. Association Between Changes in fALFF or FC and ADNI-EF

**Figure 3:**
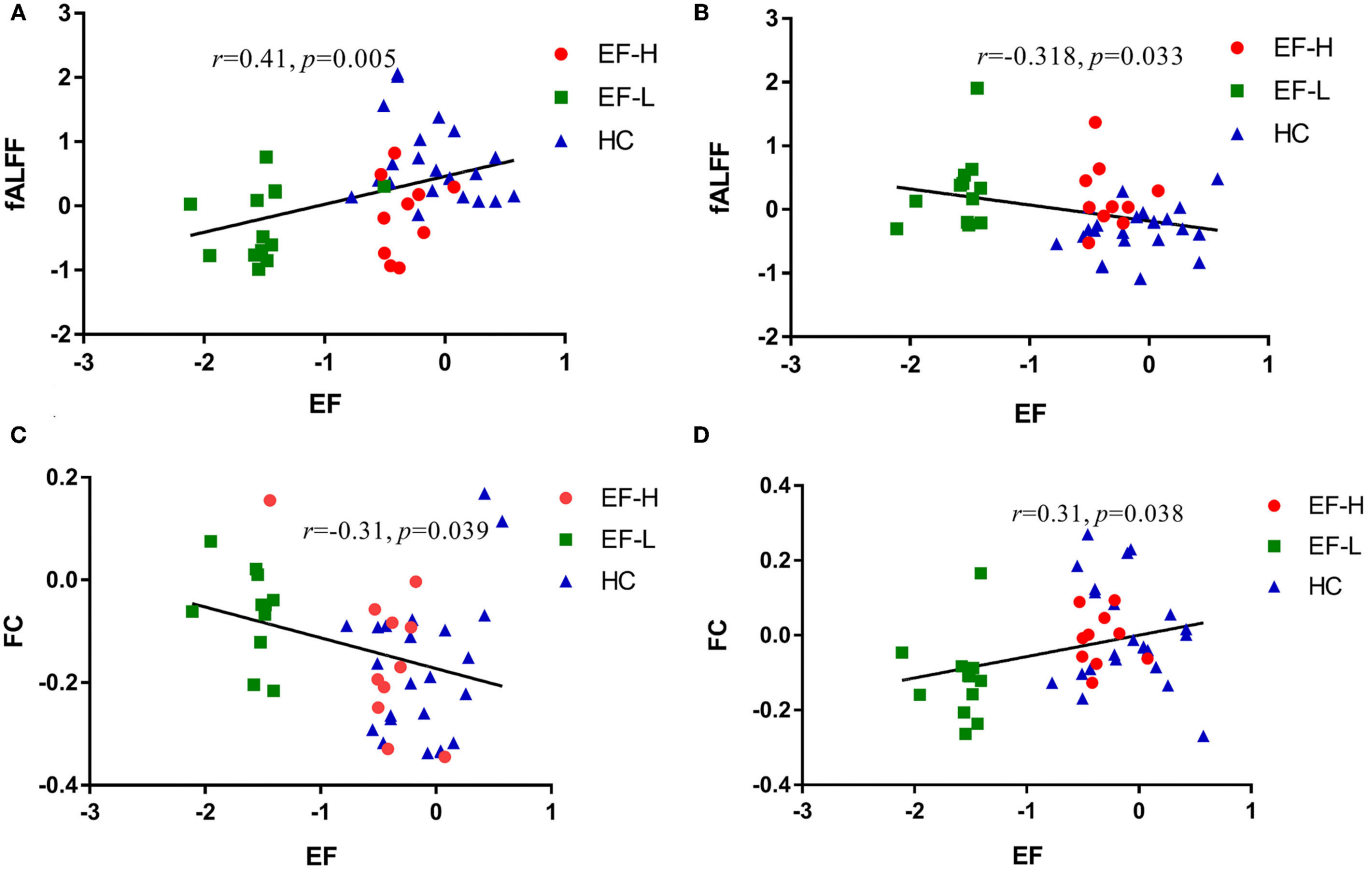
**(A)** Significant relationships between the altered fALFF in the left MFG and ADNI-EF. **(B)** Significant relationships between altered fALFF in the cerebellar vermis and ADNI-EF. **(C)** Significant relationships between altered FC in the right parahippocampal gyrus and ADNI-EF. **(D)** Significant relationships between altered FC in the left cuneus and ADNI-EF. EF-H, MCI-highEF group; EF-L, MCI-lowEF group; HC, healthy controls; EF, ADNI-EF.

Among the groups that consist of HC and MCI, the analysis demonstrated that altered fALFF in the left MFG is positively correlated to ADNI-EF (*r* = 0.41, *p* = 0.005, Figure 3A), while altered fALFF in cerebellar vermis is negatively correlated to ADNI-EF (*r* = −0.32, *p* = 0.033, Figure 3B). Altered FC between the right DLPFC and the right PHG is negatively correlated to ADNI-EF (*r* = 0.31, *p* = 0.038, Figure 3C), while altered FC between the right DLPFC and left cuneus is negatively correlated to ADNI-EF (*r* = −0.31, *p* = 0.039, Figure 3D). Age, gender, and education level are all used as covariates for these results (If Bonferroni-corrected used, the result is that only the fALLF value of the left MFG is significantly positively correlated with ADNI-EF).

## 4. Discussion

Using the fALFF and FC, we evaluated the differences in resting regional brain activity and FC of ECN among patients with MCI-highEF and MCI-lowEF subtypes. We also explored the relationship between these changes and EF. The results demonstrated that only the regional brain activity was impaired in MCI-highEF, while the FC of ECN did not change. On the other hand, not only was the regional brain activity of MCI-lowEF impaired, but the FC patterns of ECN changed. Additionally, correlation analysis indicated that altered fALFF and FC were related to impaired EF. These results suggest that the two subtypes of MCI can have different patterns of spontaneous brain activity and FC of the ECN, and the functional integrity of ECN may contribute to retained executive abilities in MCI.

Our first important finding was that the fALFF in the right precentral gyrus, right postcentral gyrus, and left MFG in the MCI-highEF group is lower than that in the HC group, while the fALFF in the left STG is higher than in the HC group. Contrastingly, the fALFF in the left MFG in the MCI-lowEF group is lower compared to HC, while fALFF of the cerebellar vermis is higher than HC. The majority of regions have been reported in prior MCI or AD studies (Cai et al., 2018; Long et al., 2018; Shi and Liu, 2020; Wang et al., 2021c). Compared to HC, the two groups all demonstrated significant fALFF differences in the left MFG. On the other hand, the frontal lobe itself was found to be an important component of ECN. A large number of previous studies have shown that the frontal lobe is closely related to cognitive function (Zhao et al., 2018; Catani, 2019; Jung et al., 2020) and EF (Cristofori et al., 2019; Zanto and Gazzaley, 2019). Herein, a study aimed to investigate the differences in atrophy patterns in the frontal-subcortical circuits between MCI and AD subjects, results of which indicated that both MCI and AD subjects had a thinner cortex in the left MFG compared to HC individuals (Zhao et al., 2015). Another structural MRI study also determined that frontal lobe atrophy was related to decreased EF in patients with aMCI (Zheng et al., 2014). Moreover, the study examined the relation between reward processing and performance on a working memory task. Results revealed that left MFG was activated by both working memory demands and increasing levels of reward (Pochon et al., 2002). Another task fMRI study showed that bilateral MFG were activated while participants performed a color-word Stroop task (Spielberg et al., 2011). Previous studies have validated that the structural and neurophysiological basis of abnormal frontal lobe spontaneous brain activity in patients with MCI, and have further verified the results of this current study. Furthermore, correlation analysis demonstrates that the fALFF of the left MFG is positively correlated to the ADNI-EF (The left MFG remained significantly positively correlated with ADNI-EF if Bonferroni-corrected was used). In other words, the lower the fALFF of the left MFG, the more severe the impairment of EF. Therefore, we hypothesize that the two groups have different patterns of spontaneous brain activity, but that the left MFG is not only a common site of injury but also closely related to EF.

In addition, our study also found that fALFF in cerebellar vermis is negatively correlated to ADNI-EF. Traditionally, the cerebellum plays an important role in the movement, maintaining body balance, regulating muscle tension, and forming voluntary movements. Thus, it was not considered to influence human cognitive function. Such an approach changed in the 1980s when research demonstrated that patients with cerebellar damage exhibited cognitive deficits (Schmahmann, 1991). Recently, much evidence has shown that the cerebellum affects not only visuospatial and verbal function, and declarative memory but also more complex behavior regulation processes, namely EF (Mak et al., 2016; Myers et al., 2017; Beuriat et al., 2020). A study found that patients with cerebellar infarction exhibited impaired cognitive function and had reduced fALFF values in the cerebellum compared to HC (Fan et al., 2019). Similarly, patients with bipolar disorder executive dysfunction showed significant hypoactivation in the cerebellum during the performance of EF tasks (Tian et al., 2020). Numerous studies have confirmed the important role of the cerebellum in EF, which is consistent with our study.

At the same time, our study indicated that the ECN connectivity pattern altered in the MCI-lowEF group, as we observed a decrease in the connection to the right PHG and an increase in the connection to the left MFG and left cuneus. However, there were no significant FC changes in the MCI-highEF group, which suggests that the functional integrity of the ECN may have contributed to retained executive abilities in MCI. More importantly, there is a positive correlation between FC and EF in the right PHG, but a negative correlation between the FC and EF in the left cuneus. Therefore, we speculate a decrease of the connection between the right PHG may be the diseased brain area related to damaging EF, while an increase in the connection of the left cuneus may be a compensatory mechanism. The PHG is known to be an important node of the hippocampal network (Zhu et al., 2020), which is vulnerable in AD for convergence of amyloid deposition, brain atrophy, functional disconnection, and hypometabolism (Sanchez et al., 2011; Trachtenberg et al., 2012). Prior studies have also shown a role of PHG in the progression of AD (Qiu et al., 2016; Wang et al., 2017). For example, some studies have demonstrated that the thickness of the PHG cortex is significantly thinner among patients with MCI (Devanand et al., 2012; Spulber et al., 2012; Machulda et al., 2020). Additionally, a longitudinal study showed that, compared to the normal control group, the converted patients with MCI showed insufficient perfusion in the right precuneus and PHG, while the MCI patients with MCI demonstrated low perfusion in the left PHG. The results of this study suggest that hypoperfusion in PHG is the earliest sign of progression from MCI to AD (Park et al., 2012). In addition, results from a meta-analysis showed the presence of significant regional resting-state differences between the aMCI and control group, which includes the posterior cingulate gyrus, right angular gyrus, right PHG, left fusiform gyrus, left supramarginal gyrus and bilateral middle temporal gyrus. The regions can be utilized as neuroimaging markers of aMCI. Thus, it can be seen that the right PHG is not only a neuroimaging marker of MCI but also a sign of progression from MCI to AD (Lau et al., 2016). This is consistent with our results and also validates that patients with MCI having low EF are more likely to progress to AD. Additionally, we identified an increase in FC in the left MFG and left cuneus. This is consistent with the majority of our previous research results, and is considered to be a compensatory mechanism.

Combining the results of fALFF and FC, both groups demonstrated the abnormal intensity of spontaneous brain activity. However, only the FC of the ECN in the MCI-lowEF group changed. This indicates that it was not just local brain regions involved, but also ECN changes with the aggravation of EF damage among patients with MCI. Executive abilities are known to require many distinct brain regions working together in order to efficiently perform complex tasks (Reineberg et al., 2018). As EF depends on global brain function, as the damage increases, the brain network needs to change. Our results further validate this point. Another important finding is that the left MFG is a commonly damaged brain area. The fALFF in the MCI-highEF group and MCI-lowEF group is lower compared to the HC group, while FC between the right DLPFC and left MFG in the MCI-lowEF group is higher compared to the HC group. Similarly, correlation analysis demonstrated that there was a positive relationship between ADNI-EF and fALFF in the left MFG (If Bonferroni-corrected was used, the left MFG remained significantly positively correlated with ADNI-EF). These results suggest that with a decrease of EF in MCI, the spontaneous brain activity of left MFG decreases, while FC of the left MFG and ECN increases, in order to compensate part of the function. At the same time, it has also been validated that the retained executive abilities in MCI are related to the functional integrity of ECN. Hence, our study provides an important and novel idea that the left MFG can be used as a target of neuroregulatory techniques for early intervention.

In addition, our current study utilized ADNI-EF to assess the EF of patients with MCI. Studies have verified that ADNI-EF is a useful comprehensive measurement of EF in MCI, as good or better as any composite part. Importantly, ADNI-EF performed the same or better than all other EF indicators in detecting changes over time, as well as in predicting dementia (Gibbons et al., 2012a). Therefore, it is more reasonable to separate patients with MCI into either the low EF group or the high EF group, according to ADNI-EF.

## 5. Limitations

Despite these results, there are still several limitations to our study. First, to ensure the authenticity of the data, we did not censor the data for matching demographics between groups, which led to significant differences in age among the three groups and, thus, may cause confusion to our results. However, in order to avoid the effect of these confounding factors, we carried out all statistical analyses with age, gender, and education level as covariates. Meanwhile, MCI - highEF group only includes two men, which will also affect our results. Therefore, we are still working on enrolling participants, and we will further validate the results after demographics matching. Second, our cross-sectional design can limit the assessment of the role of regional brain activity and changes in FC in the left MFG in the subsequent development of AD. Changes of fALFF and FC in the left MFG after the conversion of two MCI groups into AD need to be further prospectively studied. Meanwhile, differences in fALFF and FC in the left MFG between patients with MCI who will be converted into AD and those who were not converted to AD also need to be further prospectively researched. Third, the sample size in our study is small, so the results are corrected by strict multiple comparisons to ensure reliability. However, recent studies have shown that even if the results are corrected by multiple comparisons, the results of a small sample size are inconsistent. Small *P*-values may not yield robust findings (Jia et al., 2021). Therefore, we are still working on enrolling participants, and we will further validate the results when the sample size is expanded in the future.

## 6. Conclusion

Our current study demonstrates that there are different patterns of spontaneous brain activity and FC of the ECN in the MCI-highEF group and MCI-lowEF group. Furthermore, the two groups demonstrated the abnormal intensity of spontaneous brain activity, but only FC of the ECN in MCI-lowEF group changed. This suggests that not only are the local brain regions involved but also that ECN changes with the aggravation of EF damage in patients with MCI. Additionally, the functional integrity of the ECN may contribute to retained executive abilities in MCI. Furthermore, the left MFG showed synchronous abnormalities in regional brain activity and FC with peripheral brain regions, and this was positively correlated with EF. Therefore, it has been suggested that the left MFG can be utilized as a target of neuroregulatory techniques for early intervention in MCI.

## Data Availability Statement

The raw data supporting the conclusions of this article will be made available by the authors, without undue reservation.

## Ethics Statement

The studies involving human participants were reviewed and approved by Human Participants Ethics Committee of the Affiliated Brain Hospital of Nanjing Medical University (Nos. 2018-KY010-01 and 2020-KY010-02), located in Nanjing, China. The patients/participants provided their written informed consent to participate in this study.

## Author Contributions

JR, JC, and RG designed the study. XC, HG, GH, CX, WQ, WX, and SC collected the data. WL and LL analyzed the data and prepared the manuscript. All authors contributed to the article and approved the submitted version.

## Funding

This study was supported by the National Natural Science Foundation of China (No. 81701675); the key project supported by Medical Science and Technology Development Foundation, Nanjing Department of Health (No. JQX18005); the Key Research and Development Plan (Social Development) Project of Jiangsu Province (No. BE2018608).

## Conflict of Interest

The authors declare that the research was conducted in the absence of any commercial or financial relationships that could be construed as a potential conflict of interest.

## Publisher's Note

All claims expressed in this article are solely those of the authors and do not necessarily represent those of their affiliated organizations, or those of the publisher, the editors and the reviewers. Any product that may be evaluated in this article, or claim that may be made by its manufacturer, is not guaranteed or endorsed by the publisher.
