# Supplementary material for: Functional Integrity of Executive Control Network Contributed to Retained Executive Abilities in Mild Cognitive Impairment
Source: Front Aging Neurosci. 2021 Nov 26;13:710172. doi: 10.3389/fnagi.2021.710172 (PMC8664557; doi:10.3389/fnagi.2021.710172)
Supplement: Supplementary file 1 [file Data_Sheet_1.pdf]

# Supplementary Material

## 1 SUPPLEMENTARY DATA

### 1.1 NBH-ADsnp database

Data used in this study were obtained from the Nanjing Brain Hospital-Alzheimer's Disease (AD) Spectrum Neuroimaging Project (NBH-ADsnp) database (in-home website: http://192.168.8.100) (Nanjing, China). NBH-ADsnp was derived from an AD Spectrum Neuroimaging Project that was launched in January 2018 by the Institute of Brain Functional Imaging, the Affiliated Brain Hospital of Nanjing Medical University (Nanjing, China). Prof. Jiu Chen, PhD, MD, from the Affiliated B rain H ospital o f Nanjing Medical University, served as the principal investigator of NBH-ADsnp. NBH-ADsnp was initiated by Dr. Jiu Chen and Dr. Xiangrong Zhang and was named by Dr. Jiu Chen's research group (discussed by Chen Xue, Guanjie Hu, Wenwen Xu, Wan Liu, Wenzhang Qi, Siyu Wang, Jiani Xu, Shanshan Chen, and finally verified by Jiu Chen and Xiangrong Zhang).NBH-ADsnp is an observational study which includes cross-sectional and longitudinal follow-up components. The goal of NBH-ADsnp was to identify early neuroimaging biomarkers of preclinical Alzheimer's Disease (AD) spectrum subjective cognitive decline (SCD), amnestic mild cognitive impairment (aMCI), amnestic mild cognitive impairment (naMCI), and AD, to predict disease progression of individuals within the preclinical AD spectrum, and to provide imaging-based targets for individualized intervention to prevent disease deterioration from preclinical stages to the eventually progressed AD. Initially, several hundreds of elderly individuals in NBH-ADsnp, who were all Han Chinese and right-handed, were recruited from hospitals and local communities by advertising and by means of broadcasting. This database used a standardized clinical evaluation protocol that included a medical history interview, neurologic examination, a battery of neurocognitive assessments, and a resting-state MRI scan (T1, T2, 3D, DTI, and BOLD) for all participants (healthy controls (CN), SCD, naMCI, aMCI, and AD). All subjects and their study partners completed the informed consent process, and study protocol.

### 1.2 Image acquisition for the NBH-ADsnp database

MRI scanning was acquired with a Siemens 3.0-T signal scanner (Siemens, Verio, Germany) in Affiliated Brain Hospital of Nanjing Medical University. All subjects lay supine with their head fixed by foam pads with a standard birdcage head coil to minimize head movement. Participants were instructed to remain as still as possible, open their eyes remain awake, and not think of anything. High-resolution T1-weighted images were acquired by 3D magnetization-prepared rapid gradient-echo (MPRAGE) sequence (repetition time [TR] =1,900ms; echo time [TE] =2.48ms; flip angle [FA] =9 degrees; matrix =256$\times$256; field of view [FOV] =256$\times$256 mm$^2$; slice thickness/gap =1/0.5 mm; 176 slices covered the whole brain) for image registration and functional localization. The imaging took approximately 260 seconds. Functional images were subsequently collected in the same slice orientation with a gradient-recalled echo-planar imaging pulse sequence (TR= 2,000ms; TE =30ms; FA =90degrees; matrix= 64]$\times$64, FOV= 220$\times$220mm$^2$; thickness/gap =4.0/0mm;voxel size = 3.4$\times$3.4$\times$4 $mm^3$; slice numbers =36). A total of 240 volumes were obtained in this acquisition sequence and each functional resting-state session lasted 480 seconds.

**Table S1.** Head motion parameteters of mild cognitive patients and healthy controls.

| | HCs (n=23) | MCI-highEF (n=11) | MCI-lowEF (n = 14) | F($\chi^2$) | P |
|---|---|---|---|---|---|
| FD_ Power | 0.18±0.09 | 0.24±0.19 | 0.18±0.13 | 0.96 | 0.389 |
| FD_ Jenkinson | 0.09±0.05 | 0.13±0.12 | 0.10±0.08 | 0.96 | 0.390 |

Note: Data is represented by mean ± SD. Analysis of variance was utilized for FD_Power and FD_Jenkinson comparison among the three groups.
